# Quand un érythème peut sauver une vie

**DOI:** 10.11604/pamj.2014.17.147.4104

**Published:** 2014-02-28

**Authors:** Amal Taghy, Badreddine Hassam

**Affiliations:** 1 Service de Dermatologie-Vénérologie CHU Ibn Sina, Maroc; 2 Faculté de Médecine et de Pharmacie Med V Souissi, Rabat, Maroc

**Keywords:** Erythème, myélome non secrétant, cancer, Erythema, non secreting myeloma, cancer

## Image en medicine

L’érythème annulaire centrifuge n'a souvent pas de cause connue, mais on découvre parfois une cause médicamenteuse (aspirine, vaccin), infectieuse (infection streptococcique, tuberculose, mononucléose infectieuse, trypanosomiase, grippe), une dermatose chronique (pemphigus, lupus érythémateux subaigu) ou une atteinte cancéreuse distante des lésions (cancer du poumon, de la prostate, de l'utérus, leucémie, myélomes). Il se traduit par des plaques annulaires érythémateuses plus ou moins prurigineuses évoluant de manière centrifuge par poussées sur plusieurs semaines ou mois. Le traitement est uniquement étiologique quant la cause est identifiée. Nous rapportons un cas d'une patiente de 74 ans, hospitalisée un érythème annulaire centrifuge confirmé histologiquement et siégeant au niveau des faces latérales du tronc et des flancs sous forme de plaques annulaires érythémateuses, finement squameuses par endroits, prurigineuses, allant jusqu’à sept centimètres de diamètre et évoluant de façon centrifuge depuis un an. Un bilan paranéoplasique réalisé (NFS, échographie abdominale, scanner thoraco-abdomino-pelvien, FOGD, coloscopie) avait objectivé une anémie microcytaire, une ferritinémie normale et une thrombopénie, ce qui nous a amené à compléter par une BOM, un myélogramme et une électrophorèse des protéines qui ont retrouvé une prolifération plasmocytaire en faveur d'un myélome non secrétant (protéinurie de Bence Jones négative et radiographies osseuses objectivant une lyse osseuse diffuse). La patiente est actuellement hospitalisée en hématologie pour traitement étiologique.

**Figure 1:**
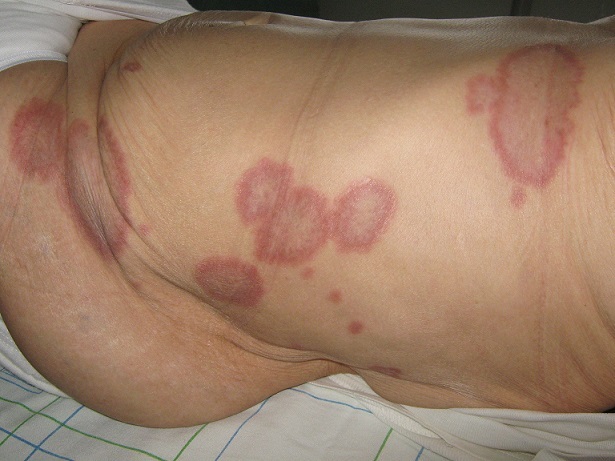
Plaquesannulaires, érythémateuses, ovalaires, bien limitées, de taille variable siégeant au niveau des flancs et des faces latérales du tronc

